# Narrative review of the COVID-19, healthcare and healthcarers thematic series

**DOI:** 10.1192/bjo.2021.1085

**Published:** 2022-02-01

**Authors:** Richard Williams, Kenneth R. Kaufman

**Affiliations:** Professor Emeritus of Mental Health Strategy, Welsh Institute for Health and Social Care, University of South Wales, UK; Presidential Lead for COVID-19, Emergency Preparedness and Mental Health to the Royal College of Psychiatrists, UK; and Director of the Psychosocial and Mental Health Programme for the Faculty of Pre-Hospital Care, Royal College of Surgeons of Edinburgh, UK; Professor of Psychiatry, Neurology and Anesthesiology, Rutgers Robert Wood Johnson Medical School, USA; and Visiting Professor, Department of Psychological Medicine, Institute of Psychiatry, Psychology & Neuroscience, King's College London, UK

**Keywords:** COVID-19, outbreaks of infectious disease, variants of SARS-CoV-2, distress, well-being, mental health disorders, the impact of COVID-19 on the public, the impact of COVID-19 on staff of healthcare services, healthcare workers (HCWs), policy, research methods, qualitative research, quantitative research, bias, noise

## Abstract

This review covers the thematic series of 22 papers selected from among manuscripts published by *BJPsych Open* concerning coronavirus disease 2019 (COVID-19) and healthcare. We report nine papers that cover concepts and epidemiology relating to the public and patients. We review 11 papers about the impact of COVID-19 on healthcare services and their staff in 15 countries. Two papers consider the psychosocial impact on staff working in mental health services in the UK. Most papers report cross-sectional analyses of data collected from convenience samples by self-reported surveys conducted at single times. They have limitations of generalisability, do not enable conclusions about diagnosis or causality, and many are likely to have attendant bias and noise. *BJPsych Open* published these papers to meet requirements for early indications of the mental health impact of COVID-19 on the public and on healthcare staff. They claim high prevalence of symptoms of anxiety, depression and post-traumatic stress. We contrast these findings with selected reports of studies with different methodologies published elsewhere. We emphasise the need for longitudinal clinical studies with refined sampling and methodological rigour. We identify several longitudinal research programmes; two in this series. We advocate tuning advice offered about caring for the public and healthcare staff to the realities of their circumstances and their perceptions of need in the context of findings from further longitudinal studies. We draw attention to the importance of the social, relationship and environmental circumstances of the public and healthcare staff in order to understand their distress and their risks of developing mental health disorders.

## Introduction

This narrative review overviews a selection of 22 papers from among those concerning coronavirus disease 2019 (COVID-19) and healthcare that *BJPsych Open* has published during the pandemic. We draw them together in this thematic series to illustrate the huge volume of literature that has been presented to many journals in the last 18 months. This series leads to several major themes and subthemes on which we comment.

Although initially planning to focus this review on the experiences and the mental health impact of COVID-19 on staff working in healthcare services, we adjusted that aim because we agree with Lamb et al, that the experiences facing healthcare staff should be seen against the backdrop of the impact on wider populations.^1^ To do otherwise ‘… prevents us from understanding whether the effect of the pandemic on their mental health is different to other key workers or the general population’.^1^ Thus, this review and our thematic series begins with illustrative papers about the general population.

Many of the papers in this thematic series focus on data collected in the early months of the pandemic, although, as time has progressed, *BJPsych Open* is receiving papers that cover a wider array of practical matters and methods. Our selection reflects a diversity of topics. We draw attention to the strengths and weaknesses of the studies conducted early in the pandemic. Importantly, these considerations lead us to emphasise the need for longitudinal studies; without them, it is difficult to adjudge their interpretation, and the severity, duration and trajectories of the impact on mental health. Advised by research on other emergencies, major incidents, conflict and outbreaks of high-consequence infectious diseases, we recognise that the effects on the mental health of the public and professionals may be delayed, develop insidiously, take time to appear and may be protracted.^2^ Jordan et al document the impact on people's health of 9/11, and we recognise the poignancy, disability and sorrow that continues to attend that event 20 years later.^2^ It reminds us that the UK National Health Service (NHS) and all healthcare services need to anticipate and be prepared for potential increased mental health demands that stem from COVID-19 over an extended time period.

Our opinion is that people's initial responses to such life-affecting events as this pandemic are very likely to represent their distress rather than mental health disorders. We require well-designed, appropriately powered and carefully executed clinical, longitudinal studies of representative samples constructed by, for example, probability sampling, to allow us to determine the nature and extent of the impact on people's mental health. Most of the research reported in the thematic series, including the mixed-methods and qualitative studies, used convenience samples. The quantitative studies used self-reported online screening questionnaires and/or psychometric scales that had been created for other research and validated previously. They provide snapshots of the impact and are illustrative of the papers that have appeared in the literature to date. In addition to the main findings, each paper presents some fascinating additional information that may have been useful in addressing the potential implications for the public and the health and social care workforce during the COVID-19 pandemic waves that have followed and in enabling the academic community to decide which risk and protective factors to research in the future. We look forward to adding papers to this selection as stronger research emerges.

We began assembling this series in April 2021 when the sheer level of fatigue of staff working in healthcare services in the UK was evident. We finished collating this series in October 2021. In the summer of 2021, pressure on staff working in the NHS in the UK started to settle somewhat although the impact of what staff had been through continued to ramify. In the UK in autumn 2021, and despite the fortitude of healthcare staff, pressures on them rose substantially again as a consequence of a virulent third wave caused by transmission of the delta variant of the SARS-CoV-2 virus; the mounting backlog of undelivered care; shortages of staff; and consequent continuing very high demands on services.^3^ These developments came as the four governments in the UK were reducing the legal enforcement of measures that were designed to reduce transmission of the virus. The omicron variant of SARS-CoV-2 arrived in the UK in November 2021; by mid-December, it had replaced delta as the dominant variant in London and it became dominant across the UK before the end of 2021. The UK's governments reintroduced measures to try to reduce transmission of the virus. The policy is that a reasonable level of personal and public protection turns on public administration of booster vaccines against a very short deadline. The spread of omicron may well curtail the third wave and usher in a fourth due to the enormously high transmissibility of the mutated virus and its greater resistance to vaccines. The measures are causing even greater pressure on the NHS and its staff.

There have been similar developments in many countries and the world situation remains precarious. With each additional variant, wave and potential restrictions, there may be varied psychosocial responses and the possibility of cumulative processes. Although longitudinal studies will best inform us about what to expect, we hope that insights gained from the initial waves and articles in this narrative review will help healthcare services and healthcarers to navigate future pandemics.

These experiences raise huge concerns about the impact of working in such challenging and risky circumstances on the mental and physical health of staff.^4^ Several papers presented online at the Trauma Care Conference in the UK in September 2021 made this point. They stir enormous gratitude and respect for the staff. We echo the sentiments of the Chief Medical Adviser to the UK Government in his presentation to the Royal College of Psychiatrists International Congress on 23 June 2021 when he recognised the enormous efforts and achievements of the staff working in healthcare services.

Table 1 summaries the papers included in the thematic series.

**Table 1:** The papers included in the thematic series (ordered by first mention in this review)

Authors	Title	Predominant research methodology	doi	Country in which data collected
Stallard et al (2021)^5^	Post-traumatic growth during the COVID-19 pandemic in carers of children in Portugal and the UK: cross-sectional online survey	Mixed methods	10.1192/bjo.2021.1	England and Portugal
Drury et al (2021)^6^	Public behaviour in response to the COVID-19 pandemic: understanding the role of group processes	Literature review	10.1192/bjo.2020.139	UK
Bartrés-Faz et al (2021)^7^	The paradoxical effect of COVID-19 outbreak on loneliness	Quantitative	10.1192/bjo.2020.163	Spain
Fancourt et al (2021)^12^	Social, cultural and community engagement and mental health: cross-disciplinary, co-produced research agenda	Qualitative	10.1192/bjo.2020.133	UK
Cobo et al (2021)^20^	Patients at high risk of suicide before and during COVID-19 lockdown: ecological momentary assessment study	Quantitative survey of a clinic sample	10.1192/bjo.2021.43	Spain
Murray et al (2021)^22^	Let us do better: learning lessons for recovery of healthcare professionals during and after COVID-19	Narrative account including a literature review	10.1192/bjo.2021.981	UK
Ben-Ezra et al (2021)^25^	Investigating the relationship between COVID-19-related and distress and ICD-11 adjustment disorder: two cross-sectional studies	Quantitative	10.1192/bjo.2020.158	UK
Chamberlain et al (2021)^28^	Post-traumatic stress disorder symptoms in COVID-19 survivors: online population survey	Quantitative	10.1192/bjo.2021.3	UK
Iqbal et al (2020)^29^	Psychiatric presentation of patients with acute SARS-CoV-2 infection: retrospective review of 50 consecutive patients seen by a consultation-liaison psychiatry team	Retrospective review of patients’ case notes	10.1192/bjo.2020.85	Qatar
Williams et al (2021)^30^	Patient adherence with infection control measures on a novel ‘COVID-19 triage’ psychiatric in-patent ward	Retrospective review of patients’ case notes	10.1192/bjo.2021.968	England
Pacchiarotti et al (2020)^37^	A psychiatrist's perspective from a COVID-19 epicentre: a personal account	Personal narrative account	10.1192/bjo.2020.83	Spain
Liu et al (2021)^40^	Psychological impact in non-infectious disease specialists who had direct contact with patients with COVID-19	Quantitative	10.1192/bjo.2020.147	China
Wanigasooriya et al (2021)^41^	Mental health symptoms in a cohort of hospital healthcare workers following the first peak of the COVID-19 pandemic in the UK	Quantitative	10.1192/bjo.2020.150	England
Hong et al (2021)^44^	Stress and psychological impact of the COVID-19 outbreak on the healthcare staff at the fever clinic of a tertiary general hospital in Beijing: a cross-sectional study	Mixed methods	10.1192/bjo.2021.32	China
Gilleen et al (2021) ^45^	Impact of the COVID-19 pandemic on the mental health and well-being of UK healthcare workers	Quantitative	10.1192/bjo.2021.42	UK
Jordan et al (2021)^46^	COVID-19 Staff Wellbeing Survey: longitudinal survey of psychological well-being among health and social care staff in Northern Ireland during the COVID-19 pandemic	Quantitative with cross-sectional and longitudinal elements	10.1192/bjo.2021.988	Northern Ireland
Yitayih et al (2021)^47^	Mental health of healthcare professionals during the early stage of the COVID-19 pandemic in Ethiopia	Quantitative	10.1192/bjo.2020.130	Ethiopia
Naldi et al (2021)^48^	COVID-19 pandemic-related anxiety, distress and burnout: prevalence and associated factors in healthcare workers of North-West Italy	Quantitative	10.1192/bjo.2020.161	Italy
Chew et al (2020)^49^	Asian-Pacific perspective on the psychological well-being of healthcare workers during the evolution of the COVID-19 pandemic	Quantitative	10.1192/bjo.2020.98	India, Indonesia, Malaysia, Singapore and Vietnam
El Abdellati et al (2021)^51^	Hospital-wide SARS-CoV-2 antibody screening of staff in a university psychiatric centre in Belgium	Quantitative	10.1192/bjo.2020.172	Belgium
Billings et al (2021)^55^	Experiences of mental health professionals supporting front-line health and social care workers during COVID-19: qualitative study	Qualitative	10.1192/bjo.2021.29	UK
San Juan et al (2021)^56^	Mental health and well-being of healthcare workers during the COVID-19 pandemic in the UK: contrasting guidelines with experiences in practice	Qualitative	10.1192/bjo.2020.148	UK

## The impact of COVID-19 on the public

### The impact of lockdown and isolation and adherence to precautionary measures

Many people have registered grave concerns about the impact of lockdown, quarantine and isolation on families and adherence of the public to the regulations whether or not people had contracted COVID-19. We include in the series three papers based on differing methodologies to draw out some potentially positive and challenging aspects of the ways in which the pandemic is managed.

First, Stallard et al report a mixed-methods study in the first wave of 385 caregivers, mainly parents, in two European countries, Portugal (*n* = 185) and the UK (*n* = 200) of whom 70% were working exclusively from home.^5^ ‘Almost half reported a reduction in income, [and] most children were home taught’.^5^ Importantly, although the cross-sectional sampling methods reported in this paper impose limitations, a single qualitative question enabled the authors to identify that nearly 90% of participants reported elements of post-traumatic growth despite their experiencing considerable adversity.

Second, Drury et al explore the literature on the role of group processes in the COVID-19 pandemic in three domains: recognition of threat; adherence by the public to the required public health behaviours; and the factors that increased such adherence during lockdown.^6^ They draw upon accumulated research on public behaviour in emergencies and the COVID-19 pandemic to show that social identity processes make better sense of the patterns of evidence than alternatives. They offer recommendations on facilitating public responses to COVID-19 by harnessing group processes.

Third, a short report by Bartrés-Faz et al documents a decrease in loneliness in 2 weeks of confinement in participants in Spain compared with their scores prior to the pandemic.^7^ Their research involved a continuing longitudinal subsample of 1604 people. It is noticeable that participants had a mean age of 55.7 years, 75% had completed higher education, and loneliness was higher before and during COVID-19 for people confined alone.

Other research suggests that we have witnessed a broad range of people's reactions to, mainly, the strictures on people's lives and employment that have evidenced a socioeconomic trajectory of greater problems experienced by less advantaged people. The UCL COVID-19 Social Study has followed 70 000 people in the UK since March 2020.^8^ That survey's researchers say, ‘The increased adherence to self-isolation rules among those with a higher household income suggests that many of those not isolating are breaking guidelines due to financial concerns …’.^8^ They have fewer choices, are more likely to have lost their jobs and have greater problems isolating in cramped housing and in coping with home schooling when they cannot fund the technology required. Fancourt et al, report elsewhere on their COVID-19 Social Study in the period March to August 2020.^9^ They include three follow-up measures completed by 36 520 participants and observed a decline in levels of anxiety and depression across the first 20 weeks after the introduction of lockdown in England. This finding emphasises the importance of longitudinal research.

Importantly, Fancourt & Bradbury's social study has found that people's experiences of the pandemic were dependent on their life situations prior to the lockdown.^10^ These findings well illustrate one of the defining features of secondary stressors.^11^ Ethnic minorities, people who are more deprived, and young people struggled much more with distress and other problems than those who are better off.^10^ These findings confirm the importance of the cross-disciplinary research agenda advocated by Fancourt et al, in an article in *BJPsych Open*, in which she and her colleagues identify the importance of focusing on social, contextual and protective factors, mechanisms of action and public engagement.^12^ Marmot et al have called for us all to ‘build back fairer’ in response to the pandemic.^13^

This situation, and the comparisons made by politicians and others early in the pandemic of its effects on populations with those of war, reminds us of similar concerns in the Second World War. The Blitz in the UK, from 1940, was forecast well before it started. Psychiatric casualties because of people's exposure to the bombing were anticipated at a ratio of three for every physical casualty,^14^ and large numbers of mental illness hospital beds were cleared to create space to which people in the UK could be admitted. In fact, few of those beds were used in this way although reviews of the history reveal the great suffering as well as the stoicism of the public and an inverse socioeconomic gradient of the impact. The way in which the UK Government's Ministry of Information used daily reports of the impact of the bombing to manage worries about public compliance contributed a partially mythical element to what is still referred to as the Blitz Spirit. In 2021, Jones contrasted the impact on the UK public of wartime aerial bombing with those of COVID-19 and the public health responses to it indicating that, by March 2021, the mortality and serious morbidity statistics were similar.^15^ Whether this comparison remains appropriate is a matter for future scrutiny. The number of deaths in the UK continued to rise in the third wave and is rising further in the fourth, and the matter of persisting serious ‘injury’ related to Long COVID or long-haul disorders is continuing to grow. Thus, as in the Second World War, the comparison turns on which issues are included.

A truth that was instrumental in how people coped in the Blitz was identified in a report on its impact on East London by Calder who observed that the thing that sustains people is ‘not letting the other fellow down and forgetting self in the common danger’.^16^ Similarly, this is a lesson we are learning again in this pandemic about sustaining the public and healthcare staff and the importance of cohesion of teams has been, and is being, emphasised recurrently.

### The impact of COVID-19 on the mental health of the public

As before the Blitz, there were claims early in the pandemic, that there would be a tidal wave of mental disorders.^1^ Many journal articles have reported that the COVID-19 pandemic has been associated with an increased frequency of a broad array of symptoms of mental disorders in the general population, mainly symptoms of anxiety, depression and post-traumatic stress. But it seems to us that the focus on these findings reflects the contents of the screening tools used rather more than an exhaustive survey of the actual array of the impact on mental health. These findings have, to the best of our knowledge, yet to be supported by substantial clinical research, but studies comprised of longitudinal surveys, structured clinical examinations and reports of people's own accounts through qualitative enquires are reaching publication or are underway.^4^ We introduce one such paper later in this review. Other studies that used cross-sectional methods but asked different questions offer a wider array of sources of anxiety.^17^ Now, 22 months after the onset of the pandemic in the UK, is still relatively early in mental illness and research terms.

It is important to identify several longitudinal studies. First, is a paper that reports research on participants in the Trøndelag Health Study in Trondheim, Norway. Participants (*n* = 2154) were recruited by repeated probability sampling to estimate prevalence of mental disorders and suicidal ideation across 2020. The authors carried out repeated cross-sectional analyses. Knudsen et al's paper supports caution about a tidal wave of impact on the mental health of the population.^18^ The authors showed that levels of mental disorders, suicidal ideation and suicide deaths were stable in the first 6 months of the pandemic and comparable with findings before the pandemic. Appleby also expressed caution based on reports from seven countries of national or state-level suicide data pointing out the suicide rates had not risen by spring 2021.^19^

In this context, a short report in our series, by Cobo et al, documents the authors’ prospective assessment of 36 adult patients in treatment in Spain because of their high risk of suicide.^20^ They administered a questionnaire using a smartphone app to attendees of a suicide-prevention out-patient clinic using an ingenious process of asking two random questions each day at random times to reduce the burden on users. They also used the Columbia Suicide Severity Rating Scale at baseline and follow-up during lockdown. The authors direct attention to two other studies that showed findings consistent with their own, that self-reported suicide risk decreased during the lockdown. It is possible, of course, that the decrease might have been temporary or an early artefact of the research and COVID-19 circumstances. We should follow these three sources to see how rates progress over time.

Ettman and colleagues report a survey conducted in March and April 2020 of 1441 respondents in the USA and compared the findings with returns from 5065 respondents from before the pandemic.^21^ They found a three-fold higher prevalence of symptoms of depression. The researchers conclude, ‘These findings suggest that there is a high burden of depression symptoms in the US associated with the COVID-19 pandemic and that this burden falls disproportionately on individuals who are already at increased risk’ in the form of having lower incomes, fewer savings and exposure to more stressors. We conclude from the works of Fancourt et al, Marmot et al and Ettman et al that there is a clear finding emerging that secondary stressors within people's life circumstances are having profound effects on the risks run by populations of their developing symptoms of mental disorders and that these stressors may be exacerbated by events associated with the pandemic. This is a good illustration of how secondary stressors operate.^11^ Murray et al also make this point and we return to that paper later.^22^

Another paper considers the longitudinal trajectories of the public's mental health responses to the COVID pandemic in the UK.^23^ Pierce et al, report a secondary analysis of the large UK Household Longitudinal Study that has been collecting data since 2009. The assessment covered the period April to October 2020 and used the 12-item General Health Questionnaire (GHQ-12) to assess mental health in 19 763 adults. This research showed that most people remained resilient, or their mental health returned to pre-pandemic levels after a deterioration in the population mean in April 2020. ‘One smaller group of people had sustained elevated scores (4.1% of the cohort) and another group (7.0% of the cohort) had little deterioration …’ but showed a steady decline in mental health over time. This paper also addresses predictors of deterioration of this nature and coming from an ethnic minority background, pre-existing mental illness, financial adversity and SARS-CoV-2 infection emerged as risk factors, as they have in other research. Pierce et al remind us of research on trajectories of psychosocial impact after major incidents that has documented a number of patterns of change over time of people's experiences or symptoms of stress and distress.^24^ The two most common patterns or longitudinal ‘trajectories’ are: (a) mild–stable levels of distress; and (b) decreasing symptoms from moderate to mild over months and years. Smaller proportions of people affected by major incidents may show: (c) deteriorating patterns of worsening stress; or (d) high-stable levels of stress. These last two groups appear more at risk of psychopathology.

We include in this thematic series two papers that report public prevalence data for possible mental health disorders.^25,28^ Both these papers are based on self-reported symptoms measured using screening questionnaires given to cross-sectional convenience samples. The response rates were either not stated or low.

The first, by Ben-Ezra et al, reports two studies of 1293 and 1073 UK participants in the early weeks of the pandemic.^25^ In the first, the authors used random stratified sampling to construct an internet panel and the K6 to screen for ‘serious mental illness’. The response rate for study 1 was 37% and it found 16.6% of the participants who reported COVID-19-related social life or occupationally stressful events to have elevated levels of symptoms of serious mental illness. The second study was intended to replicate the findings from the first. The authors report an elevated risk of serious mental illness and probable ICD-11 adjustment disorder among people who say they have had stressful events related to COVID-19. Both studies showed stratified increased risk based on number of stressful events. This is an interesting finding because it resonates with the ‘cumulative risk model’. This model is the dominant thesis regarding children stating that ‘… long-term adverse outcomes are better predicted by the total number, rather than the specific nature of environmental risk exposures’ to ‘… different adverse experiences and events’ early in childhood.^26,27^

In the second paper, Chamberlain et al report a study that examined, in May 2020, post-traumatic stress disorder (PTSD) symptoms in 13 049 people in the UK aged 16 or older who self-reported suspected or confirmed COVID-19, predominantly from the UK general population.^28^ Participants were recruited through television webpages. This project identified escalating effect sizes with increased severity of COVID-19 and degree of treatment.

Research by Iqbal et al aimed to ascertain the psychiatric morbidity associated with SARS-CoV-2 infection by a retrospective study of case notes of 50 consecutive patients for whom psychiatric diagnoses reflected clinical judgement.^29^ Having COVID-19 was operationally defined as patients having ‘… a positive antigen test for SARS-CoV-2 [real-time polymerase chain reaction test] taken during the patient's current period of hospital admission’. All were in-patients in hospitals in Doha, Qatar who were referred to a consultation-liaison team. The authors report that a psychiatric diagnosis was made in 49 cases and that ‘… a third of our sample had a past psychiatric history, and of these nearly half had a psychotic disorder or bipolar I disorder …’. The authors say that this ‘… suggests that those with prior mental health problems are particularly vulnerable to develop further mental health problems during the pandemic’.

Another retrospective case-notes study, by Williams et al, reports patients’ adherence with measures taken by an acute psychiatric in-patient ward in London to control spread of SARS-CoV-2 among patients.^30^ The authors report that 138 of 176 patients did not comply with the measures and that people who had diagnoses of psychotic, personality and substance use disorders were less adherent than those with other disorders. Their adherence improved when they were given direct instructions. We recognise that there are many potential weaknesses in studies that use case notes to glean data retrospectively but this one shows the importance of giving patients clear instructions about protecting themselves and other people.

It has also become clear that many people, including healthcare staff, have developed sustained healthcare problems of a broad nature that stem from embolic and other phenomena that have been caused by the SARS-CoV-2 virus and the immune responses to it. The National Institute for Health Research in England is publishing reviews of ‘long COVID’.^31^ Paterson describes a spectrum of COVID-19 neurology,^32^ Baig documents deleterious outcomes in ‘long-haulers’ who have a chronic COVID syndrome as a result of SARS-CoV-2,^33^ and Nalbandian et al, provide an account of persistent and prolonged effects after acute COVID-19.^34^ Hypotheses for understanding the origins, and treatments for these persisting effects of COVID-19 are presently emerging. One important observation is that the occurrence of long-haul problems is not necessarily related to the severity of COVID-19 experienced by patients.^35^

## The impact of the pandemic on healthcare services and their staff

As Lamb et al noted in 2020, ‘Anyone working in the health service at present has likely noticed … a proliferation of surveys on health-care workers.’^1^ By the spring of 2021, staff in the NHS in the UK were shattered, whether or not they had patient-facing jobs, although staff in more intense and senior management roles were especially so. This is likely to be similar in healthcare systems across the world. There can be no doubt about the degrees of stress and distress that staff have experienced and are experiencing. Murray et al offer a review of how the demands have been experienced by healthcare staff in the UK.^22^ They indicate that distress may result from primary stressors i.e. those sources of stress that are inherent in the pandemic including the risks to their own health and their fear of transmitting the virus to patients, relatives and colleagues. Also, we recognise that some health and social carers’ experiences might result from the COVID-19 disease processes that can cause psychiatric symptoms.

From spring to autumn 2021, levels of fatigue among UK healthcare staff were almost palpable.^3^ In mid-December 2021, healthcare staff were moving from tackling the third wave in the UK, to dealing with demands caused by the omicron variant. Measures to slow the new variant's transmission have been introduced to find time to deliver a very demanding plan to offer booster vaccinations to the whole of the UK's adult population in a very short timescale. As we have observed, the demands on healthcare staff have been enormous. But now there are even greater pressures and NHS England declared a new Level 4 national incident and suspended some routine work in the light of the activity required by the vaccination programme and preparations for the potential significant increase in COVID-19 cases.

During autumn 2021, there were proportionately fewer hospital admissions in the UK, consequent on the country's vaccination status. As we enter a fourth wave, the circumstances regarding hospital admissions and deaths as a result of the omicron variant are uncertain. This raises huge concern about the public, healthcare services but also about the impact on staff working in such challenging and risky circumstances for their mental and physical health, while also stirring enormous gratitude and respect for their selfless efforts.

At times, conspiracy theorists have suggested that the extent of the pandemic has been exaggerated and, even, that its existence is a hoax.^22^ These claims have been experienced as hurtful by healthcare staff who have put themselves and their families at risk and have worked and are working long and hard to care for the public. Some have contracted COVID-19, no small number of staff has died, and some have contracted Long COVID.

Murray et al,^22^ describe how the distress experienced by so many staff may also arise from secondary stressors. They are defined in Williams et al as: ‘1. Social factors and people's life circumstances (including the policies, practices, and social, organisational, and financial arrangements) that exist prior to, but impact them during the major incident, emergency, disaster, conflict, or disease outbreak; and/or 2. Societal responses to the major incident or emergency’.^11^ They may exacerbate primary stressors. They include matters that may be secondary to coping with the existence of COVID-19 and its ramifications, for example, telecommuting and remote education with decreased social contact. Although the secondary stressors might appear to be more trivial, our experiences show that some concerning longer-term matters such as access to showers, hot food and parking spaces have had a huge impact on staff during the pandemic.^11,22^ But chief of all these concerns among staff has been reliable access to personal protective equipment (PPE) that meets the standards set by the World Health Organization.

In summary, Murray et al draw attention to the importance of understanding that COVID-19 is having an impact on an NHS in the UK that was already chronically stressed, and this raises the importance of understanding the balance of primary and secondary stressors that staff faced and continue to face, and finding effective ways to support them. Furthermore, previous research on other types of major incident and emergencies has identified two broad pathways in which groups of people, first, mobilise support although, later, that support deteriorates.^36^ Murray et al observed both the support mobilisation and support deterioration pathways among the public and staff working in the NHS during the first wave.^22^

### The impact of COVID-19 on staff working in healthcare

Our thematic series includes a personal account of the opening 6 months of the pandemic as it affected services and their staff in Spain. Pacchiarotti et al, describe how Spain reconfigured its hospitals and redeployed healthcare professionals to manage demand early in the pandemic.^37^ They say that these actions led to healthcare staff being exposed to extremely stressful situations and the authors projected ‘… a rebound effect on mental health problems … in the medium and long term …’. They called for preventive approaches, use of telepsychiatry and evaluation of altered ways of providing mental healthcare. We have seen each of these proposals in action in the past 18 months.

An important matter concerns the risks to staff who address the ethical dilemma of choosing who receives services when healthcare systems are overwhelmed. Triage and triage-like decision-making remains with people who make those decisions for a substantial time afterwards.^38^ Thus, the potential for staff to experience moral distress and moral injury have been widely discussed in the UK though the pandemic. Murray et al introduce both moral distress and moral injury.^22^ Rimmer summarises claims made by the British Medical Association after its survey of doctors conducted in March and April 2021.^39^ She says that around half of responders had heard of moral distress and moral injury and 78% of responders (around 1800 doctors) said that moral distress resonated with their experiences at work whereas 51% said the same for moral injury. Doctors who worked only with patients who had COVID-19 were more likely to give these responses as were doctors from ethnic minority backgrounds compared with doctors who are White.

Two papers in the thematic series reinforce the psychosocial importance of PPE availability with associated workplace training,^40,41^ and these concerns continued through the second wave in the UK. In March 2021, the Royal College of Nursing published an independent report that found that the UK's guidelines for ventilating healthcare premises and face protection had not been updated to reflect evidence about airborne transmission of SARS-CoV-2.^42^

Early in the pandemic, Kisely et al, reported a systematic review of the psychological effects of what are now known as high-consequence infectious diseases (HCIDs) on healthcare workers over the past 20 years.^43^ They found that the organisational measures that best decrease the risk of adverse outcomes include: positive feedback to staff; the faith of staff in local infection control procedures; providing protective gear; effective preparation; and training.

### Research into the impact on the mental health of healthcare staff

The thematic series includes eight papers that describe surveys, mainly online, of the impact of the pandemic on healthcare staff conducted in a number of countries using self-administered screening tools with convenience samples.^40,41,44–49^ We identify a mix of primary and secondary stressors emerging from these papers as associated with participants’ experiences of distress and symptoms of mental health disorders. Seven of the papers in the thematic series (dates of sampling in parenthesis) report findings from a single country; two papers are from China (March 2020;^40^ February to March^44^); three from the UK (June to July 2020;^41^ April to May 2020^45^ and November 2020 time 1, February 2021 time 2^46^); one from Ethiopia (March 2020^47^); and one from Italy (March 2020^48^). The eighth paper reports on healthcare staff in India, Indonesia, Malaysia, Singapore and Vietnam (April to June 2020^49^). These papers offer differing but helpful snapshots of the mental health of staff in the first wave of the pandemic and all report high levels of psychiatric symptoms.

In the first paper from China, Liu et al report mental health symptoms experienced by obstetricians and midwives who were not infected as they treated hospital in-patients who had COVID-19.^40^ The authors found high rates of symptoms of depression (42%), anxiety (29%) and insomnia (34%) in a total sample of 2126 participants. ‘Regardless of whether or not they had direct contact with patients with COVID-19, obstetricians and midwives were more likely to report mild and moderate depression and anxiety during the COVID-19 pandemic when compared with before the pandemic’. Higher rates of symptoms of depression and insomnia were reported by staff who had direct contact with patients with COVID-19. ‘Those who had sufficient protective equipment or training were less likely to report depression, anxiety and insomnia than those who did not’.^40^

Hong et al report a cross-sectional study of the psychological impact of the COVID-19 pandemic on the healthcare staff working in a fever clinic of a general hospital in Beijing using qualitative interviewing, and the Sources of Stress questionnaire (developed during the SARS outbreak in Hong Kong) and the Impact of Event Scale-Revised (IES-R) questionnaire.^44^ There were 102 participants, (39% doctors; 53% nurses and 8% technicians) who stayed in the fever clinic during their rotation, and adjustments were made to their working conditions to help them adapt to the work. Staff received semi-structured interviews from staff of a hotline support service and the records were coded by scenario analysis and some answers were converted to binary variables. The total score on the Sources of Stress correlated moderately with the IES-R score. The top four sources of distress were worry about: the health of one's family or others; the spread of the virus; changes in work; and one's own health.

Wanigasooriya et al report on an electronic survey of hospital healthcare workers in the West Midlands, UK using clinically validated questionnaires.^41^ ‘The rates of … clinically significant symptoms of anxiety, depression and PTSD were 34.3%, 31.2% and 24.5%, respectively’. The factors that emerged as negatively correlated with participants reporting symptoms included availability of adequate PPE and well-being support, and lower exposure to moral dilemmas at work.

Gilleen et al, looked at the impact of the pandemic on the well-being and mental health of healthcare workers in the UK.^45^ They endeavoured to: quantify how the mental health of healthcare workers changed compared with before the pandemic; identify if healthcare workers on the front line, based in London or from ethnic minorities, had more severe symptoms compared with other staff; quantify the prevalence of severe psychiatric symptoms; and identify factors associated with those symptoms. They conducted a Qualtrics web-based survey open to all UK healthcare workers that presented the nine-item Patient Health Questionnaire (PHQ-9), seven-item anxiety scale (GAD-7), IES-R and Perceived Stress Scale to participants. Only healthcare workers ‘… who had experienced a stressful or traumatic event related to COVID-19 were administered the IES-R. Also, the Connor-Davidson Resilience Scale (CD-RISC) was administered’. ‘Nearly a third of HCWs [healthcare workers] reported moderate to severe levels of anxiety and depression; and the number reporting very high symptoms was more than quadruple that pre-COVID-19’ (albeit measured retrospectively on Likert scales). Factors that influenced the results were reported to cluster within the themes of: demographics and roles; workplace readiness; risk management including PPE; experience of stressful events; protective experiences including sharing stress at work. The authors report that ‘Front-line workers were significantly more likely to be more depressed, anxious, have high PTSD symptoms and be more stressed than non-front-line workers…Working in London was associated with lower risk of depression … and anxiety … than outside London (although there was no difference in stress or PTSD). Ethnic minority status (*n* = 342) was significantly associated with greater risk of high PTSD symptoms (OR [odds ratio] = 1.52), but not high anxiety, stress or depression’. ‘Being able to share stress at work … [was] associated with significantly lower likelihood of being in the high anxiety, stress and depression groups …’.^45^

Jordan et al report findings at two of four time points for which analyses are complete in an ongoing, online cross-sectional and longitudinal survey of the prevalence of mental health symptoms affecting health and social care staff in Northern Ireland.^46^ Data collection at time 1 was in November 2020 (3834 staff responded, response rate 4.9%) and at time 2 was in February 2021 (2898 responses, response rate 3.7%). The validated questionaries were the GAD-7, PHQ-9, IES-R and Insomnia Severity Index. As the survey was open to new respondents at time 2, the authors report the initial findings as two cross-sectional studies. A high proportion of staff reported moderate-to-severe symptoms of depression, anxiety, PTSD and insomnia in the cross-sectional samples used at time 1 (26 to 30%) and time 2 (27 to 36%). Significantly more participants reported symptoms suggesting moderate-to-severe depression at time 2 but the other symptoms were not significantly different compared with time 1. These cross-sectional findings, which appeared to Jordan et al higher compared with findings from other studies of general UK and Irish populations, were ‘broadly mirrored’ in their longitudinal analyses comparing findings at times 1 and 2. But the authors also draw attention to another paper that found no significant differences between the prevalence rates for healthcarers and other populations.^50^

A hospital-based cross-sectional study was conducted in Ethiopia with 249 healthcare professionals by Yitayih et al.^47^ The authors report the ‘prevalence of psychological distress among healthcare professionals was 78.3% … [and prevalence] of insomnia was 50.2%’. Interestingly, we note that higher rates of psychological distress were associated with not having a daily update on COVID-19 and feeling stigmatised or rejected because of working in a hospital.

Naldi et al, report a cross-sectional, survey of 797 healthcare workers in North-West Italy.^48^ This study used the IES-R to measure distress, the State-Trait Anxiety Inventory (Form Y) to measure anxiety and the Maslach Burnout Inventory. The authors found that one-third of their participants had severe anxiety and distress. They draw attention to higher rates of distress in women and nurses. The authors conclude that detecting factors ‘associated with worst psychological outcome may favour a tailored, preventive, organisational and psychological approach, representing a potential strength in counteracting the effects of future pandemics’.

Chew et al, report on a total of 1146 participants from India (384), Indonesia (250), Malaysia (175), Singapore (277) and Vietnam (60).^49^ They used the Depression Anxiety Stress Scales (DASS-21) and IES-R to measure symptoms. Their samples included doctors, nurses, administrative staff, pharmacists, cleaners, porters and technicians. The authors conclude that their study shows that ‘the varied prevalence of psychological adversity among healthcare workers is independent of the burden of COVID-19 cases within …’ each of the countries studied. Interestingly, the authors say that Vietnam, which had the lowest volume of cases of COVID-19 per day per million population, also had ‘a higher prevalence of PTSD related to COVID-19 among healthcare workers compared with India’. Participants in Singapore ‘reported the highest number of cases per day/1 million, but had a lower prevalence of depression and anxiety among its healthcare workers, when compared with the Malaysian cohort’. Consistent with the opinions of other researchers, the authors of this paper comment on the potential beneficial effects from early psychological interventions for vulnerable groups of healthcare workers. These findings support our contention that other factors in addition to the risks introduced by COVID-19 were involved, as suggested by Fancourt et al and others.^8–10,22^

### A serosurvey of the staff working in mental health services

One paper in the thematic series reports the first published serosurvey among psychiatric healthcare providers showing a lower seroprevalence compared with the results from other studies in the general population and other healthcare workers in Belgium.^51^ This paper reports 3.2% of psychiatric providers were seropositive,^51^ whereas the proportion of staff in a tertiary medical centre were reported as 6.4%.^52^ Exposure at home predicted the presence of antibodies, but exposure at work did not. The authors recommend measures to prevent transmission from staff to patients in psychiatric facilities.

## Caring for and supporting staff working in healthcare services

Another question raised by the thematic series is how should we develop reasoned approaches to caring for, and supporting healthcare staff? Murray et al suggest using a framework published before the pandemic^22,53^ as a way of understanding and responding to the many psychosocial and mental health effects of the pandemic on staff.^53^ These interventions fall into the categories of: supporting the well-being of every member of staff (the Wellbeing Agenda); providing focused psychosocial interventions to meet the needs of staff who are struggling or have become distressed (the Psychosocial Agenda); while remaining aware that a smaller number of staff may develop conditions that require specialist mental health assessment and, possibly, treatment (the Mental Health Agenda). This fits with the findings in the papers we summarise and this approach to caring for staff is now included in guidance for the NHS in England from NHS England and NHS Improvement.^54^

The first wave of the COVID-19 pandemic was attended by huge public solidarity and mutual support in the UK. That support extended to staff in the NHS for whom the public clapped on Thursday evenings.^22^ But before the end of the first wave, cohesion had begun to falter, and the clapping stopped. Anecdotally, over the summer of 2020, UK staff began to take in the realities of what they had been through and became fatigued and/or disillusioned.^22^ Then came the second wave. Staff began to report anecdotally the difficulties in finding the energy and determination required to ‘go again’. Clearly, coping with the second and third waves has been a very different experience compared with the first.^22^

Many mental health providers have reached out to their colleagues and they, in turn, have come under strain. In this regard, the paper by Billings et al, is highly relevant.^55^ The research team conducted qualitative interviews with 28 mental health professionals from varied professional backgrounds, career stages and across the UK who supported front-line healthcare staff. Many of them experienced professional growth. However, this came with the costs of additional responsibilities and increased workloads; many were professionally isolated and were affected vicariously by the experiences that healthcare workers talked about in interviews. Plainly, staff who support their colleagues also require care and support. A vital point to which this paper draws our attention is who cares for the carers?

At the beginning of the pandemic, many professional people were eager to express solidarity with their colleagues working at the front line by preparing guidance on how they should protect their psychosocial and mental health. But was this guidance helpful? San Juan et al, used qualitative methods to interview 33 front-line healthcare workers in the UK to look at the applicability of 14 sets of well-being guidelines in practice and provide recommendations for supporting front-line staff during the current and future pandemics.^56^ The authors found that many of the guidelines emphasised the importance of staff receiving psychological support and avoiding mental health disorders, whereas the healthcare workers placed greater emphasis on structural conditions at work, responsibilities outside the hospital and the support of the community (once again, secondary stressors). Similarly, Jordan et al draw attention to the consistency of their findings about the ‘importance of organisational factors to staff well-being’.^46^ Thus, Murray et al and other authors identify the importance of peer support.^22,57^ But the supporting interventions proposed in the guidelines reviewed by San Juan et al ‘did not always respond to the lived experiences of staff, as some reported not being able to participate because of understaffing, exhaustion or clashing schedules’.^56^ The authors concluded that ‘Well-being guidelines should explore the needs of healthcare workers, and contextual characteristics affecting the implementation of recommendations’.^56^ Although reporting a relatively focused study, this paper does show a way forward in meeting the needs of staff; the findings support the importance of community engagement and co-production in agreeing how to offer support.^12^

## Research methods and understanding the findings

The papers in the themed series report studies conducted by a variety of research methods. Three papers report studies in which the authors used predominantly qualitative methods, 11 papers report the results of predominantly quantitative epidemiological methods, and the data in 2 papers derived from mixed methods. One paper offers a personal account, two are based on literature or narrative reviews, two offer findings from reviews of patients’ case notes and one paper reports quantitative research on a clinic sample. Two of the papers used longitudinal approaches.

All the papers report methods and sampling processes that preclude conclusions as to actual levels of diagnosed conditions or causality. In their paper, Jordan et al recognise some of the sampling challenges raised by studying restricted numbers of healthcare staff rather than representative samples of the whole workforce and raise the importance of longitudinal research.^46^ Although providing useful pictures of symptoms experienced at, usually, a single time point, most of the quantitative studies in the series are based on cross-sectional analyses of data collected from convenience samples by self-report using screening tools. The limitations of these methods regarding the generalisability of the findings and making interpretations of causality are evident. Questionnaires and screening tools cannot be diagnostic and, especially when self-completed, tend to overestimate rates of disorders.^1^ Knudsen et al, make similar points when they make assertions about the importance of explicit sampling frames using known populations.^18^ Some, but relatively few, of the studies we present here have control populations or multiple sampling points. Also, many of the studies were conducted in the first months of the pandemic and have used short recruitment periods.

*BJPsych Open* published early papers as ‘snapshots’ or harbingers of what might be psychological and psychiatric concerns associated with the pandemic. A number of those papers identify what we call secondary stressors as important matters that have an impact on risk.^11^ This topic is raised in the paper by Murray et al who show its importance.^22^ Many of the studies reported in the papers in the series use additional questionnaires to explore factors that might bear on the experiences of staff working in healthcare services. The results from these questionnaires are very interesting and they serve as initial but incomplete presentations of pertinent stressors. We are tempted to draw from some of them impressions of the apparently positive adjustments of many healthcare staff based on these wider enquiries that contrast with the rates of symptoms that the screening tools indicate. This might fit with the possibility that participants were reporting distress and departures from well-being in self-reported surveys rather than symptoms of disorders. Necessarily, questionnaires control the discourse between participants and researchers, but participants may be tempted to fit their own experiences to the fixed questions asked. This suggests to us that mixed-methods research using both qualitative and quantitative approaches are required when researching novel experiences such as those the public and staff have faced in this pandemic.

The methodological limitations we cover also raise the importance of bias and noise in the investigations that further limit the conclusions that can be drawn from many of these studies. Biases, whether conscious or unconscious, tend to systematically shift sample means left or right in a distribution of scores whereas noise creates wider variability of scores within the distribution. Noise may occur within participants and between participants and we guess that ‘level’, ‘pattern’ and ‘occasion’ variations in scores that constitute noise are likely to occur in self-reported surveys.^58^

Another important methodological matter concerns the researchers’ choices of instruments. It is important to be aware that choice of measures in studies is likely to reveal the researchers’ assumptions about the definitions of distress and disorders and other features such as resilience. We opine that greater clarity of definitions, and greater rigour in explaining choice of measures are required.

We conclude that most of the papers in our series may well describe a surge in distress, but we are unclear about levels of both persistent distress and rates of disorders and the methods used do not allow us to distinguish distress from disorders. Smith, Editor-in-Chief of *The Lancet*, makes a similar point about distinguishing distress from disorder.^59^ It is also, for example, possible that more staff took part in some of the studies because they were concerned about their mental health and were, possibly, unwell.

Knudsen et al^18^ point out that chronic stressors and economic recession may be factors that have a long-term impact on the mental health of the public. In this context, the papers in the thematic series present data from, mainly, the first wave of the pandemic. We recognise that the early impact may be rather different compared with the longer-term impact, and we know from research into other contrasting events, such as 9/11, just how long some people's serious mental health disorders may go on and how delayed may be their presentations.^2^ Therefore, it is important to monitor research conducted during subsequent waves and for many years afterwards. We are aware, for example, that research on paramedics and emergency medical technicians involved on 9/11 into the long-term impact on their physical and mental health bear out this matter.^60^ The study by Smith & Burkle was conducted in 2016 – 15 years after 9/11; it documents the seriousness of the enormous long-term mental and physical health effects on ambulance staff of their participation in caring for people involved in that event.^60^ In the short-term, we should be clear that distress is not equivalent to mental health disorders. In the longer-term, studies of distress, mental health and physical health disorders are needed to examine whether there is deterioration of public mental and physical health consequent on the pandemic.

Nonetheless, although *BJPsych Open* recognises the, arguably, inevitable limitations of many studies conducted early in the pandemic, it also recognised at the time that there was a desire for initial knowledge and, consequently, published a number of papers to offer readers access to initial findings. Our longer-term goal is to include longitudinal studies with comparators to better define the development of symptoms, disorders and causality as time elapses and opportunities arise in and after the pandemic. Therefore, we emphasise the need for longitudinal and clinical research studies over a much greater time that incorporate comparisons with similar groups pre-pandemic or that use matched controls.

## Conclusion

A huge amount of research has been done very rapidly in the pandemic. We are eager to bring together this thematic issue because it illustrates the papers that have been presented *to BJPsych Open* that offer a picture of the ways in which populations and healthcare workers have been affected in the opening months of a much longer-term emergency. They claim high prevalence of symptoms of anxiety, depression and post-traumatic stress. Jordan et al remark that the prevalence figures from their work with health and social care staff are higher than those from studies of the public. But they also draw attention to a paper from Cénat et al that found no significant differences between the prevalence rates for healthcarers and other populations.^46,50^ The methods used in many studies do not allow us to form any definite conclusions. We, as Knudsen et al and Appleby, express caution.^18,19^ But pragmatically, we are learning a huge amount about the value of, and best methods for supporting the public and our colleagues. There can be no doubt of the importance of relationships in that task as a number of the papers point out. One of the papers identifies that post-traumatic growth is another possibility.^5^

As the fourth wave continues in the UK, we remember that other countries may be in different phases. Even if the COVID-19 pandemic can be brought under control through vaccination across the world, and that will take a long time yet, we must face the economic effects that create chronic stressors that may lead to mental health problems. This is the time to pursue more research and to employ methods that are appropriate to addressing long-term distress, mental health symptoms and disorders, protective and risk factors, and the development and treatment of long-term neuropsychiatric sequelae of COVID-19. The papers included here have another important function; they inform us about refining research methods with a view to producing more definitive learning.

## Data Availability

Data from the published papers to which this article refers can be obtained, as indicated, from the authors for each referenced paper; the authors of this narrative review do not have data to share.
